# Sex-specific differences in healthcare utilization and preventive service uptake between immigrant and native-born populations in Spain: a matched analysis of the 2023 national health survey

**DOI:** 10.3389/fpubh.2026.1894084

**Published:** 2026-08-05

**Authors:** Ana Lopez-de-Andres, Jose J. Zamorano-Leon, Rodrigo Jimenez-Garcia, David Carabantes-Alarcon, Natividad Cuadrado-Corrales, Andres Bodas-Pinedo, Clara Maestre-Miquel, Ana Jimenez-Sierra, Lucia Fuentes-Arroyo

**Affiliations:** 1 Department of Public Health and Maternal & Child Health, Faculty of Pharmacy, Universidad Complutense de Madrid, Instituto de Investigación Sanitaria del Hospital Clínico San Carlos (IdISSC), Madrid, Spain; 2 Department of Public Health and Maternal & Child Health, Faculty of Medicine, Universidad Complutense de Madrid, Instituto de Investigación Sanitaria del Hospital Clínico San Carlos (IdISSC), Madrid, Spain; 3 Department of Nursing, Physiotherapy and Occupational Therapy, Faculty of Health Sciences, University of Castilla-La Mancha, Talavera de la Reina, Spain; 4 Faculty of Medicine, Universidad CEU San Pablo, Madrid, Spain

**Keywords:** health inequalities, healthcare utilization, immigrants, preventive services, Spain

## Abstract

**Objective:**

To assess sex-specific differences in healthcare utilization and preventive service uptake between immigrant and native-born populations in Spain in 2023, and to determine whether these differences persist after adjustment for sociodemographic characteristics, comorbidities, and lifestyle factors.

**Methods:**

We conducted a population-based cross-sectional study using data from the 2023 Spanish National Health Survey. The study included 5,044 participants (2,522 immigrants and 2,522 native-born individuals) selected through a 1:1 matched design according to sex, age, and autonomous community of residence. Healthcare utilization (primary care, specialist consultations, psychology, physiotherapy, dental care, emergency visits, and hospitalization) and adherence to preventive activities (cancer screening, influenza vaccination, cholesterol testing, and blood glucose testing) were analyzed. Analyses were stratified by sex. Conditional logistic regression models estimated adjusted odds ratios (ORs) and 95% confidence intervals (95%CIs), progressively adjusting for sociodemographic variables, comorbidities, and lifestyle-related factors.

**Results:**

Immigrant men and women showed lower utilization of specialist and outpatient services, including psychologist, physiotherapy, and dental care, with lower participation in preventive activities, particularly cancer screening and influenza vaccination. No differences were observed in primary care use, emergency department visits, or hospitalizations. These differences were more pronounced among women. After full adjustment, immigrant status was not associated with primary care utilization, emergency visits, or hospital admissions. However, native-born men and women showed higher adjusted odds of outpatient service utilization, including specialist consultations (men: OR 1.28; women: OR 1.31), psychologist visits (men: OR 1.56; women: OR 1.96), physiotherapy (men: OR 2.06; women: OR 2.04), and dental care (men: OR 1.39; women: OR 1.32). Native-born participants also showed higher adjusted odds of colorectal cancer screening, cholesterol testing, and blood glucose testing, whereas immigrant women had lower odds of breast and cervical cancer screening and influenza vaccination.

**Conclusion:**

Immigrant populations in Spain exhibit lower healthcare utilization and preventive service uptake than native-born individuals. However, these differences were partly attenuated after adjustment for sociodemographic characteristics, health status, and lifestyle factors, suggesting that these factors contribute to the observed disparities. Inequalities were especially pronounced in preventive care among women, highlighting the need for gender-sensitive, culturally tailored strategies addressing social determinants to achieve equitable healthcare utilization.

## 1 Introduction

International migration has become a major demographic phenomenon with important implications for population health and the organization of healthcare systems. Understanding how immigrant populations interact with healthcare systems is therefore essential from both clinical and public health perspectives (1, 2).

A growing body of research has examined differences in healthcare utilization between immigrant and native-born populations. Overall, immigrants tend to use certain healthcare services less frequently, particularly specialist care and preventive services, while utilization of primary care and emergency services appears more heterogeneous (3–5). However, findings across studies are not entirely consistent. While some analyses report lower healthcare utilization among immigrants, others observe similar or even higher use once demographic, socioeconomic, and health-related factors are considered (5). These inconsistencies suggest that healthcare utilization patterns among immigrants are shaped by multiple interacting determinants rather than access barriers alone.

Preventive healthcare is a particularly relevant domain in this context, as lower participation of immigrant populations in preventive activities has been consistently documented even in health systems with universal coverage (3, 6).

Gender further shapes healthcare utilization and preventive behaviors. Although women usually have higher participation in preventive activities than men (7), the intersection between migration and gender remains insufficiently explored. Available evidence suggests that migration-related barriers may interact with gender-specific determinants of health, potentially amplifying inequity, particularly in preventive care (2, 6).

Spain provides a relevant setting to examine these issues because the Spanish National Health System offers universal coverage even for immigrants in irregular situation. Although previous studies have consistently reported differences in healthcare utilization between immigrant and native-born populations in Spain, several important knowledge gaps remain (3, 6, 8–21). Much of the available evidence is based on surveys conducted before the COVID-19 pandemic or on administrative databases that lack detailed information on socioeconomic characteristics, self-rated health, health behaviors, and other social determinants of health. Consequently, their ability to comprehensively evaluate the factors associated with healthcare utilization is limited. Moreover, previous Spanish studies have generally focused on a limited number of healthcare services, most commonly primary care, specialist consultations, or emergency department use, without providing a comprehensive assessment across the continuum of care. Little attention has been paid to services that are predominantly delivered through the private healthcare sector in Spain, such as dental care, physiotherapy, and psychological care, where barriers to utilization may differ from those operating in publicly funded services. Preventive healthcare has also frequently been examined separately from healthcare utilization, despite representing an essential component of equitable healthcare access. Finally, few nationwide studies have simultaneously adopted a matched, sex-stratified design with extensive adjustment for sociodemographic characteristics, comorbidities, and lifestyle factors, limiting understanding of whether observed disparities persist after accounting for differences in health needs and population characteristics (3, 4, 6, 8–21).

Therefore, the aim of this study was to assess sex-specific differences in healthcare utilization and preventive service uptake between immigrant and native-born populations in Spain in 2023, and to examine is associations remained after adjustment for sociodemographic characteristics, comorbidities, and lifestyle factors.

## 2 Material and methods

### 2.1 Study design and data source

We conducted a population-based cross-sectional study using data from the 2023 Spanish National Health Survey (SNHS 2023). This survey was jointly developed by the Spanish Ministry of Health and the National Statistics Institute (INE) and targets the non-institutionalized population residing in Spain aged 15 years and over. Data collection was carried out from September 2023 to August 2024.

The SNHS 2023 uses a probabilistic, stratified, three-stage sampling design. In the first stage, census sections were selected as primary sampling units. In the second stage, households (main family dwellings) were randomly selected within each section. In the third stage, one adult per household was selected using the Kish method. This design ensures representativeness at both national and regional levels (22).

### 2.2 Study population and matching procedure

Participants were classified according to their country of birth. Individuals born outside Spain were considered immigrants, whereas those born in Spain were classified as native-born.

A matched analytical design was applied. Each immigrant participant was matched 1:1 with a native-born individual by sex, age, and autonomous community of residence. Matching by autonomous community of residence was performed to minimize potential confounding arising from regional differences in socioeconomic context, healthcare organization, service availability, and health policies across Spain.

### 2.3 Study variables

The main study variable was country of birth, obtained from the question “What is your country of birth?” and categorized as immigrant (born outside Spain) or native-born (born in Spain).

The dependent variables included healthcare utilization and adherence to preventive health activities.

Healthcare utilization variables referred to the use of services in the previous 12 months and included: visits to a primary care physician, specialist consultations, psychologist visits, physiotherapist visits, dentist visits, emergency room visits, and at least one hospital admission.

Preventive activities were also assessed. Breast cancer screening was evaluated by asking participants whether they had undergone a mammogram within the previous 3 years, considering women aged 40–69 years as eligible. Cervical cancer screening was assessed based on self-reported Pap smear within the previous 3 years among women aged 25–65 years. Colorectal cancer screening adherence was determined by the performance of a fecal occult blood test (FOBT) within the previous 2 years, considering men and women aged 50–69 years as eligible.

Additionally, other preventive practices included influenza vaccination in the last campaign and screening tests such as cholesterol and blood glucose measurements within the previous 12 months.

Covariates included sociodemographic characteristics such as educational level, employment status, living with a partner, and private health insurance. Private health insurance was included as an indicator of socioeconomic position because, in Spain, it is strongly associated with greater economic resources. Household income was not included because of its low response rate in the survey. Lifestyle-related factors collected were body mass index, self-rated health, smoking status, alcohol consumption, and physical activity. The body mass index was categorized in obesity, overweight and normal. Finally, the self-reported presence of physicians diagnosed relevant comorbidities (cardiovascular diseases, musculoskeletal diseases, respiratory diseases, gastrointestinal disorders, neurological disorders, mental disorders, cancer, diabetes and hypertension) was analyzed. The definitions and categorization of all variables are detailed in Supplementary Table 1.

### 2.4 Statistical analysis

All analyses were stratified by sex. A descriptive analysis of the study population was performed. Quantitative variables were expressed as mean and standard deviation (SD) when normally distributed, or as median and interquartile range (IQR) when non-normally distributed, based on the Kolmogorov–Smirnov test. Categorical variables were described as frequencies and percentages.

Comparisons between immigrant and native-born participants were conducted separately for men and women. The chi-square test was used for categorical variables, while the Student's *t*-test or Mann–Whitney U test was applied for continuous variables, as appropriate.

To assess the association between country of birth and healthcare utilization and preventive activities according to gender, conditional logistic regression models were fitted, taking into account the 1:1 matched design (matched by sex, age, and autonomous community of residence). Separate models were constructed for each dependent variable.

Four sequential models were developed: Model 1 was adjusted for age; Model 2 was additionally adjusted for sociodemographic variables (educational level, employment status, living with a partner, and private health insurance); Model 3 included adjustment for comorbidities and lifestyle-related factors (body mass index, self-rated health, smoking status, alcohol consumption, physical activity, and chronic conditions); and Model 4 was fully adjusted, including all covariates. Only those variables with significant association in the bivariate analysis or considered relevant according to the bibliography review, were included in the models.

The strength of the associations was expressed as odds ratios (OR) with their corresponding 95% confidence intervals (95% CI). Statistical significance of the variables was assessed using the Wald test.

Multicollinearity was evaluated using the variance inflation factor (VIF), with values < 2 considered indicative of no relevant multicollinearity. Model calibration was assessed using the Hosmer–Lemeshow goodness-of-fit test.

All analyses were performed using Stata statistical software (StataCorp, College Station, TX, USA). A two-sided *p*-value < 0.05 was considered statistically significant.

### 2.5 Ethical considerations

The data used in this study were obtained from the Spanish National Health Survey (SNHS 2023), which is publicly available through the official website of the Spanish Ministry of Health (23). The dataset is fully anonymized and does not contain any personal identifiable information.

The use of anonymized, publicly available data ensures compliance with current data protection regulations. Therefore, in accordance with Spanish legislation on secondary data analysis, neither ethical approval nor informed consent was required for this study.

## 3 Results

A total of 5,044 participants were included, comprising 2,522 immigrants and 2,522 matched native-born individuals. Of these, 2,876 (57.0%) were women. The mean age of the study population was 45.36 years (SD, 15.1).

Table 1 shows the comparison of sociodemographic, clinical and lifestyle characteristics between immigrant and native-born men. Compared with native born men, immigrant men had a significantly lower educational level, with a higher proportion having completed their studies before the age of 13 years (17.6% vs. 7.3%) and a lower proportion with university education (22.1% vs. 27.9%; *p* < 0.001). Employment status differed significantly (*p* < 0.001), with higher unemployment among immigrants (12.8% vs. 8.5%) and higher prevalence of retirement or disability among native-born men (16.4% vs. 12.6%). Immigrant men were more likely to live with a partner (53.8% vs. 47.9%; *p* = 0.006). No differences were observed in private health insurance coverage or self-rated health.

**Table 1:** Sociodemographic characteristics, comorbidities, and lifestyle factors among men according to country of birth in Spain (SNHS 2023).

Variables	Categories	Immigrant	Native–born	*p*–value
		*N* (%)	*N* (%)	
Educational level	Ended before 13 years of age:	183 (17.6)	77 (7.3)	<0.001
	Ended from 13 to 16 years:	233 (22.5)	256 (24.1)	
	Ended from 16 to 18 or vocational training	392 (37.8)	433 (40.8)	
	Completed university studies	229 (22.1)	296 (27.9)	
Employment status	Working	753 (70.0)	744 (69.1)	<0.001
	Unemployed	138 (12.8)	91 (8.5)	
	Retired or disable	135 (12.6)	176 (16.4)	
	Homework or studying	49 (4.6)	65 (6.0)	
Living with a partner	Yes	576 (53.8)	507 (47.9)	0.006
Private health insurance coverage	Yes	113 (10.5)	132 (12.3)	0.192
Self–rated health	Very good, good	858 (79.2)	847 (78.1)	0.564
Any chronic condition	Yes	447 (41.8)	568 (53.0)	<0.001
High blood pressure	Yes	178 (16.4)	199 (18.4)	0.234
Heart diseases and stroke	Yes	67 (6.2)	73 (6.7)	0.600
Musculoskeletal diseases	Yes	189 (17.4)	226 (20.8)	0.043
Respiratory disease	Yes	69 (6.4)	103 (9.5)	0.007
Gastrointestinal disorders	Yes	102 (9.4)	130 (12.0)	0.052
Neurological disorders	Yes	47 (4.3)	75 (6.9)	0.009
Mental health disorders	Yes	74 (6.8)	123 (11.3)	<0.001
Cancer	Yes	17 (1.6)	22 (2.0)	0.419
Diabetes	Yes	56 (5.2)	69 (6.4)	0.231
Alcohol use	Dailly or weekly	343 (32.0)	480 (45.0)	<0.001
	Few times a year	337 (31.4)	380 (35.6)	
	Not last year or ever	392 (36.6)	206 (19.3)	
Tobacco use	Currently	256 (23.7)	307 (28.5)	<0.001
	Ex smoker	182 (16.9)	257 (23.9)	
	Never smoked	641 (59.4)	513 (47.6)	
Physically active	Yes	305 (28.6)	446 (41.9)	<0.001
Body mass index	Normal	413 (39.4)	433 (41.3)	0.521
	Overweight	490 (46.7)	464 (44.2)	
	Obesity	146 (13.9)	152 (14.5)	

Immigrant men showed a lower overall prevalence of chronic conditions (*p* < 0.001), including musculoskeletal, respiratory, neurological and mental disorders, while no significant differences were observed for hypertension, cardiovascular disease, diabetes, gastrointestinal disorders or cancer. Lifestyle patterns also differed significantly, with lower alcohol consumption (weekly/daily: 32.0% vs. 45.0%), lower prevalence of current smoking (23.7% vs. 28.5%), and lower physical activity (28.6% vs. 41.9%; all *p* < 0.001) among immigrant men. BMI did not differ between groups.

Table 2 illustrates healthcare utilization and preventive practices among men according to country of birth. No significant differences were found in primary care use (approximately 70% in both groups), emergency department visits or hospitalizations. In contrast, immigrant men had lower use of specialist care (45.5% vs. 52.0%; *p* = 0.002), psychologist visits (4.1% vs. 8.2%; *p* < 0.001), physiotherapy (10.6% vs. 24.4%; *p* < 0.001) and dental services (41.3% vs. 52.7%; *p* < 0.001). Preventive activities also differed, with lower participation among immigrants in colorectal cancer screening (FOBT: 31.9% vs. 42.5%; *p* = 0.004), cholesterol testing (55.6% vs. 61.8%; *p* = 0.003) and blood glucose testing (50.8% vs. 56.8%; *p* = 0.005), while influenza vaccination did not differ.

**Table 2:** Healthcare utilization and preventive activities among men according to country of birth in Spain (SNHS 2023).

Variables	Categories	Immigrant	Native–born	*p*–value
		*N* (%)	*N* (%)	
Primary care doctor visit in last 12 months	Yes	765 (70.6)	788 (72.7)	0.273
Specialist doctor visit in last 12 months	Yes	493 (45.5)	564 (52.0)	0.002
Psychologist visit in last 12 months	Yes	44 (4.1)	89 (8.2)	<0.001
Physiotherapist visit in last 12 months	Yes	115 (10.6)	264 (24.4)	<0.001
Dentist visit in last 12 months	Yes	448 (41.3)	571 (52.7)	<0.001
Emergency room visit in last 12 months	Yes	191 (17.6)	222 (20.5)	0.090
Hospital admission in last 12 months	Yes	62 (5.7)	69 (6.4)	0.528
FOBT last 24 months (aged 50–69 years old)	Yes	111(31.9)	148(42.5)	0.004
Influenza vaccine in last season	Yes	182 (16.8)	189 (17.4)	0.690
Blood cholesterol in last 12 months	Yes	603 (55.6)	670 (61.8)	0.003
Blood sugar in last 12 months	Yes	551 (50.8)	616 (56.8)	0.005

FOBT, Fecal occult blood test.

Table 3 shows the comparison of sociodemographic, clinical and lifestyle characteristics between immigrant and native-born women. Immigrant women had lower educational attainment, with more reporting studies completed before age 13 (15.7% vs. 7.4%) and fewer reporting university education (25.4% vs. 38.2%; *p* < 0.001). Employment status differed significantly (*p* < 0.001), with lower employment (53.8% vs. 63.4%) and higher unemployment among immigrants (16.1% vs. 10.9%). Immigrant women were less likely to have private health insurance (13.0% vs. 16.7%; *p* = 0.005). No differences were observed in living with a partner or self-rated health.

**Table 3:** Sociodemographic characteristics, comorbidities, and lifestyle factors among women according to country of birth in Spain (SNHS 2023).

Variables	Categories	Immigrant	Native–born	*p*–value
		*N* (%)	*N* (%)	
Educational level	Ended before 13 years of age:	216 (15.7)	104 (7.4)	<0.001
	Ended from 13 to 16 years:	273 (19.8)	271 (19.4)	
	Ended from 16 to 18 or vocational training	538 (39.1)	488 (35.0)	
	Completed university studies	350 (25.4)	533 (38.2)	
Employment status	Working	769 (53.8)	900 (63.4)	<0.001
	Unemployed	230 (16.1)	154 (10.9)	
	Retired or disable	176 (12.3)	194 (13.7)	
	Homework or studying	254 (17.8)	171 (12.1)	
Living with a partner	Yes	685 (48.7)	661 (47.6)	0.562
Private health insurance coverage	Yes	185 (13.0)	239 (16.7)	0.005
Self–rated health	Very good, good	993 (69.1)	1,039 (72.3)	0.060
Any chronic condition	Yes	778 (54.9)	853 (60.7)	0.002
High blood pressure	Yes	239 (16.6)	202 (14.0)	0.056
Heart diseases and stroke	Yes	64 (4.5)	76 (5.3)	0.298
Musculoskeletal diseases	Yes	393 (27.3)	401 (27.9)	0.739
Respiratory disease	Yes	136 (9.5)	140 (9.7)	0.800
Gastrointestinal disorders	Yes	165 (11.5)	196 (13.6)	0.081
Neurological disorders	Yes	213 (14.8)	216 (15.0)	0.875
Mental health disorders	Yes	197 (13.7)	294 (20.4)	<0.001
Cancer	Yes	52 (3.6)	56 (3.9)	0.695
Diabetes	Yes	80 (5.6)	54 (3.8)	0.021
Alcohol use	Dailly or weekly	269 (18.9)	402 (28.3)	<0.001
	Few times a year	507 (35.6)	584 (41.0)	
	Not last year or ever	649 (45.5)	437 (30.7)	
Tobacco use	Currently	172 (12.0)	317 (22.2)	<0.001
	Ex smoker	154 (10.8)	310 (21.7)	
	Never smoked	1,106 (77.2)	802 (56.1)	
Physically active	Yes	259 (18.1)	460 (32.4)	<0.001
Body mass index	Normal	687 (50.1)	804 (58.8)	<0.001
	Overweight	468 (34.1)	389 (28.4)	
	Obesity	216 (15.8)	175 (12.8)	

Immigrant women had a lower overall prevalence of chronic conditions (54.9% vs. 60.7%; *p* = 0.002) and mental disorders (13.7% vs. 20.4%; *p* < 0.001), but a higher prevalence of diabetes (5.6% vs. 3.8%; *p* = 0.021). As in men, immigrant women reported lower alcohol consumption, smoking prevalence and physical activity (all *p* < 0.001). In contrast to men, immigrant women showed a less favorable body mass index distribution, with higher prevalence of overweight and obesity (*p* < 0.001).

Table 4 presents healthcare utilization and preventive practices among women. No differences were observed in primary care use (79.9% vs. 81.5%), emergency department visits or hospitalizations. Immigrant women showed lower use of specialist services (57.0% vs. 65.5%; *p* < 0.001), psychologist visits (5.8% vs. 14.0%; *p* < 0.001), physiotherapy (12.7% vs. 26.9%; *p* < 0.001) and dental care (49.2% vs. 61.1%; *p* < 0.001).

**Table 4:** Healthcare utilization and preventive activities among women according to country of birth in Spain (SNHS 2023).

Variables	Categories	Immigrant	Native–born	*p*–value
		*N* (%)	*N* (%)	
Primary care doctor visit in last 12 months	Yes	1,149 (79.9)	1,172 (81.5)	0.277
Specialist doctor visit in last 12 months	Yes	820 (57.0)	942 (65.5)	<0.001
Psychologist visit in last 12 months	Yes	84 (5.8)	201 (14.0)	<0.001
Physiotherapist visit in last 12 months	Yes	183 (12.7)	387 (26.9)	<0.001
Dentist visit in last 12 months	Yes	707 (49.2)	878 (61.1)	<0.001
Emergency room visit in last 12 months	Yes	356 (24.8)	362 (25.2)	0.796
Hospital admission in last 12 months	Yes	97 (6.7)	75 (5.2)	0.084
Mammogram in last 36 months (aged 40–69 years old)	Yes	428 (56.5)	517 (68.2)	<0.001
Pap Smear in last 36 months (aged 25–65 years old)	Yes	659 (56.9)	816 (70.4)	<0.001
FOBT last 24 months (aged 50–69 years old)	Yes	112 (27.9)	145 (36.2)	0.013
Influenza vaccine in last season	Yes	261 (18.2)	335 (23.3)	<0.001
Blood cholesterol in last 12 months	Yes	819 (57.0)	919 (63.9)	<0.001
Blood sugar in last 12 months	Yes	769 (53.5)	839 (58.3)	0.009

FOBT, Fecal occult blood test.

Participation in preventive activities was also lower among immigrant women, including breast cancer screening (56.5% vs. 68.2%), cervical cancer screening (56.9% vs. 70.4%), colorectal cancer screening with FOBT (27.9% vs. 36.2%), influenza vaccination (18.2% vs. 23.3%), cholesterol testing (57.0% vs. 63.9%) and blood glucose testing (53.5% vs. 58.3%).

Tables 5, 6 show the results of conditional logistic regression analyses for healthcare utilization and preventive activities among men and women, respectively. After full adjustment (model 4), country of birth was not associated with primary care utilization, emergency visits or hospitalizations in either sex. In contrast, native-born men showed higher odds of specialist consultations (OR 1.28; 95% CI 1.02–1.59), psychologist visits (OR 1.56; 95% CI 1.03–2.38), physiotherapy use (OR 2.06; 95% CI 1.55–2.74) and dental care (OR 1.39; 95% CI 1.13–1.71). Native-born women exhibited a comparable pattern, with higher odds of specialist consultations (OR 1.31; 95% CI 1.08–1.60), psychologist visits (OR 1.96; 95% CI 1.37–2.82), physiotherapy use (OR 2.04; 95% CI 1.59–2.62) and dental care (OR 1.32; 95% CI 1.10–1.60).

**Table 5:** Conditional logistic regression models for healthcare utilization and preventive activities among men according to country of birth in Spain (Immigrant men as the reference group).

Variables	Model 1	Model 2	Model 3	Model 4
	OR (95%C)	OR (95%C)	OR (95%C)	OR (95%C)
Primary care doctor visit in last 12 months	1.13 (0.92–1.39)	1.17 (0.93–1.47)	1.05 (0.82–1.35)	1.04 (0.82–1.32)
Specialist doctor visit in last 12 months	1.31 (1.10–1.56)	1.31 (1.08–1.59)	1.25 (1.02–1.52)	1.28 (1.02–1.59)
Psychologist visit in last 12 months	2.12 (1.45–3.09)	1.82 (1.21–2.73)	1.82 (1.23–2.70)	1.56 (1.03–2.38)
Physiotherapist visit in last 12 months	2.60 (2.04–3.30)	2.60 (2.00–3.37)	2.00 (1.52–2.63)	2.06 (1.55–2.74)
Dentist visit in last 12 months	1.60 (1.34–1.90)	1.52 (1.25–1.84)	1.50 (1.22–1.83)	1.39 (1.13–1.71)
Emergency room visit in last 12 months	1.20 (0.97–1.48)	1.17 (0.93–1.48)	1.08 (0.84–1.40)	1.06 (0.84–1.34)
Hospital admission in last 12 months	1.12 (0.78–1.59)	1.03 (0.71–1.51)	1.05 (0.69–1.60)	1.01 (0.67–1.50)
FOBT in last 24 months (aged 50–69 years old)	1.66 (1.19–2.31)	1.50 (1.06–2.14)	1.43 (0.97–2.10)	1.52 (1.05–2.18)
Influenza vaccine in last season	1.06 (0.81–1.40)	1.01 (0.75–1.35)	0.98 (0.71–1.35)	0.97 (0.70–1.34)
Blood cholesterol in last 12 months	1.34 (1.11–1.61)	1.32 (1.08–1.62)	1.26 (1.01–1.57)	1.26 (1.01–1.59)
Blood sugar in last 12 months	1.30 (1.09–1.56)	1.32 (1.09–1.61)	1.32 (1.06–1.63)	1.35 (1.09–1.66)

Model 1: crude; Model 2: Model 1 + adjusted by sociodemographic variables; Model 3: Model 1 + adjusted by comorbidity and lifestyle; Model 4: adjusted by all variables.

FOBT, Fecal occult blood test.

**Table 6:** Conditional logistic regression models for healthcare utilization and preventive activities among women according to country of birth in Spain. (Immigrant women as the reference group).

Variables	Model 1	Model 2	Model 3	Model 4
	OR (95%C)	OR (95%C)	OR (95%C)	OR (95%C)
Primary care doctor visit in last 12 months	1.11 (0.92–1.34)	1.04 (0.84–1.29)	1.06 (0.83–1.34)	1.07 (0.84–1.36)
Specialist doctor visit in last 12 months	1.45 (1.24–1.69)	1.29 (1.08–1.53)	1.34 (1.11–1.62)	1.31 (1.08–1.60)
Psychologist visit in last 12 months	2.62 (2.00–3.44)	2.54 (1.89–3.42)	1.78 (1.27–2.49)	1.96 (1.37–2.82)
Physiotherapist visit in last 12 months	2.53 (2.07–3.09)	2.40 (1.92–3.02)	2.06 (1.61–2.63)	2.04 (1.59–2.62)
Dentist visit in last 12 months	1.61 (1.38–1.87)	1.42 (1.20–1.69)	1.39 (1.16–1.66)	1.32 (1.10–1.60)
Emergency room visit in last 12 months	1.02 (0.86–1.21)	0.96 (0.79–1.16)	0.91 (0.73–1.13)	0.92 (0.73–1.15)
Hospital admission in last 12 months	0.75 (0.54–1.03)	0.75 (0.54–1.04)	0.78 (053–1.16)	0.79 (0.54–1.14)
Mammogram in last 36 months (aged 40–69 years old)	1.76 (1.40–2.21)	1.61 (1.24–2.09)	1.69 (1.28–2.24)	1.59 (1.21–2.09)
Pap Smear in last 36 months (aged 25–65 years old)	1.83 (1.53–2.19)	1.61 (1.31–1.97)	1.61 (1.29–1.99)	1.60 (1.27–2.02)
FOBT in last 24 months (aged 50–69 years old)	1.48 (1.09–2.01)	1.41 (1.02–1.95)	1.51 (1.08–2.11)	1.43 (1.01–2.04)
Influenza vaccine in last season	1.47 (1.20–1.81)	1.44 (1.15–1.81)	1.42 (1.11–1.81)	1.34 (1.04–1.72)
Blood cholesterol in last 12 months	1.39 (1.18–1.63)	1.34 (1.12–1.60)	1.32 (1.08–1.60)	1.36 (1.10–1.67)
Blood sugar in last 12 months	1.24 (1.06–1.45)	1.27 (1.05–1.47)	1.34 (1.11–1.62)	1.34 (1.12–1.62)

Model 1: crude; Model 2: Model 1 + adjusted by sociodemographic variables; Model 3: Model 1 + adjusted by comorbidity and lifestyle; Model 4: adjusted by all variables.

FOBT, Fecal occult blood test.

Regarding preventive activities, native-born men showed higher adjusted odds of colorectal cancer screening (FOBT) (OR 1.52; 95% CI 1.05–2.18), cholesterol testing (OR 1.26; 95% CI 1.01–1.59) and blood glucose testing (OR 1.35; 95% CI 1.09–1.66). Similar associations were observed among native-born women, who also showed higher odds of colorectal cancer screening (OR 1.43; 95% CI 1.01–2.04), blood cholesterol testing (OR 1.36; 95% CI 1.10–1.67) and blood glucose testing (OR 1.34; 95% CI 1.12–1.62). In addition, native-born women presented higher odds of breast cancer screening (OR 1.59; 95% CI 1.21–2.09), cervical cancer screening (OR 1.60; 95% CI 1.27–2.02) and influenza vaccination (OR 1.34; 95% CI 1.04–1.72).

## 4 Discussion

This study provides a comprehensive and updated assessment of sex-specific differences in healthcare utilization and participation in preventive services between immigrant and native-born populations in Spain in 2023. Overall, immigrant populations showed lower use of healthcare services and lower engagement in preventive activities compared with native-born individuals. However, these differences were attenuated after accounting for sociodemographic, clinical, and lifestyle characteristics, suggesting that observed disparities reflect a complex interplay between healthcare needs, population composition, and structural determinants rather than differences in access alone. These patterns were observed in both sexes, although differences were particularly pronounced among women, especially in preventive activities such as cancer screening and vaccination.

The observation that differences in healthcare utilization and preventive practices were partly reduced after adjustment for individual characteristics is consistent with previous evidence and underscores the importance of contextualizing utilization patterns within broader social and health frameworks. Rather than representing a simple indicator of differential access to care, these disparities could be affected by many other factors such as variations in population structure, baseline morbidity, social position, and health needs (24, 25). This interpretation is particularly relevant in universal healthcare systems, where formal coverage does not necessarily ensure equitable use of services.

Evidence from Spain supports our results. Population studies show immigrants do not use healthcare more than natives and often use it less (8–12). Data from the Spanish National Health Survey 2006 found lower or similar use among most immigrant groups, varying by care level, origin, and sex (9). Later surveys confirmed these patterns over time, including during the economic crisis, with persistent differences across care levels (8, 10). The Spanish National Health Survey 2017 likewise showed lower use across socioeconomic groups, while emergency care use was similar (11). Consistently, Henares-Montiel et al. found no major differences in primary or emergency care, but more unmet need among immigrants, especially men (12). Comparable patterns have been reported across Europe. A systematic review found migrants generally use fewer preventive services, while specialist care use is more variable and sometimes similar or higher (5).

Importantly, our results show that inequalities in healthcare use are not uniform but concentrated in specific services, including specialist, mental health, physiotherapy, dental care and health prevention services. Common possible explanations for these disparities are the combined effects of language barriers, lower health literacy, unfamiliarity with the organization of the healthcare system, cultural beliefs and health-seeking behaviors, and socioeconomic disadvantages. Financial constraints may be particularly relevant for healthcare services that are predominantly delivered through the private sector in Spain, where out-of-pocket payments or private insurance are often required. In addition, precarious employment, inflexible working hours, and caregiving responsibilities may further reduce the ability to attend scheduled appointments, particularly for services requiring proactive engagement or repeated visits (24–27). Subsequent evidence shows that gaps partly disappear after accounting for health needs and social determinants, indicating that disparities reflect population composition more than access barriers (2, 27). Studies under similar access conditions support this, showing marked attenuation after adjustment for morbidity and sociodemographic factors (13, 28).

Our results should also be viewed considering the healthy immigrant effect, whereby immigrants often show better health than the host population, especially soon after arrival (29, 30). In Spain, Gotsens et al. reported an initial advantage that declined over time and during the economic crisis (10). Post-crisis data suggest a partial recovery, with the European Health Survey in Spain 2014 showing indicators consistent with a renewed effect (12). However, the healthy immigrant effect tends to deteriorate over time also due to chronic stress, social exclusion, precarious living conditions and barriers to health care access (29), which contribute to the development of the Ulysses syndrome, also known as the Syndrome of the Immigrant with Chronic and Multiple Stress (30).

Consistently, Villarroel and Artazcoz found lower specialist use among immigrants, varying by sex and origin, while primary care differences were limited (9). The persistence of lower specialist care utilization after full adjustment suggests that barriers extend beyond differences in health status or healthcare needs. Specialist consultations generally require referral through primary care, scheduled appointments, and greater navigation of the healthcare system than primary care services. In Spain, prolonged waiting times in the public sector and the possibility of obtaining faster access through private healthcare may further widen these differences, as immigrants are less likely to have private health insurance or the financial resources to pay for specialist consultations. Together, these organizational and financial barriers may contribute to the persistent inequalities observed in specialist care utilization (9, 13, 21).

Lower use of mental health services among immigrants has been widely reported and warrants attention (5, 14, 29, 31). Disparities in mental health service utilization are likely to reflect a combination of structural and cultural factors. Unlike many other healthcare services, psychological care relies heavily on effective communication and trust between patients and providers, making language barriers particularly relevant (29, 31). Furthermore, stigma surrounding mental illness, cultural perceptions of emotional distress, and limited knowledge of available mental health services may discourage professional help-seeking (31). In Spain, these barriers may be compounded by long waiting times within the public mental healthcare system and the widespread use of private psychological services, which may be less affordable for immigrant populations. Importantly, lower utilization should not be interpreted as lower need, as previous evidence suggests that immigrants may experience substantial unmet mental healthcare needs despite lower service use (29, 31).

Mental health stigma is a barrier to psychological care in both native-born and immigrant populations (32, 33). However, systematic reviews consistently report that stigma plays a disproportionately important role among immigrants, whereas previously mentioned, it frequently coexists with cultural beliefs, language barriers, limited mental health literacy, and distrust of healthcare systems, contributing to lower utilization of psychological services despite similar or greater mental health needs (32, 33).

As described by other authors dental care showed one of the largest and most persistent disparities, which is consistent with the financing model of oral healthcare in Spain (15, 16, 34). Because routine adult dental care is predominantly provided through the private sector, access depends largely on the ability to pay. Lower income, limited private insurance coverage, and competing financial priorities may therefore discourage routine dental attendance among immigrants. In addition, preventive dental care may receive lower priority than other immediate social and economic needs, resulting in delayed utilization until symptoms become severe (15, 16, 34).

A key finding of our investigation is the consistently lower use of preventive services among immigrants, especially women. Differences were seen in cancer screening and influenza vaccination, with the largest gaps in breast and cervical screening. Lower uptake also appeared in colorectal screening and overall vaccination, indicating a consistent pattern. This aligns with international evidence showing reduced use of preventive services among immigrants (35, 36).

Evidence across Europe shows that immigrant women are less likely to undergo breast and cervical cancer screening than native-born women, with differences persisting after adjustment for age and sometimes socioeconomic factors (35–37). Similar patterns are seen in colorectal cancer screening, with lower participation in fecal occult blood testing among immigrants across Europe, showing disparities extend beyond sex-specific programs (38). Influenza vaccination shows similar inequalities: coverage is lower among immigrants (39).

The inequalities observed in preventive screening among immigrant women probably reflect barriers that differ from those influencing healthcare utilization for symptomatic conditions. Participation in screening programs requires proactive engagement with the healthcare system and depends on understanding the purpose of screening, responding to invitations, and attending appointments despite the absence of symptoms (35–39). Language barriers, lower health literacy, unfamiliarity with organized screening programs, cultural beliefs regarding cancer and gynecological examinations, and competing work and caregiving responsibilities may all contribute to lower participation. The finding that these disparities remained significant despite extensive adjustment suggests that universal availability of screening programs alone may be insufficient to ensure equitable participation (35–40). We also cannot exclude the possibility that differences in reproductive history and contact with maternity services may have influenced some preventive healthcare practices among women, as information on pregnancy and prenatal care was not available in the survey.

The lower use of cholesterol and blood glucose testing among immigrants deserves consideration because these investigations are generally requested in primary care. One possible explanation is a lower perceived clinical need, as immigrants in our study generally reported better health and fewer chronic conditions. However, these tests also represent opportunistic preventive care and are commonly requested during routine health assessments rather than consultations for acute problems. Therefore, the observed differences may also reflect fewer opportunities for preventive assessment rather than reduced access to primary care itself (21). Further studies are needed to clarify whether these findings reflect differences in consultation patterns, clinical decision-making, or uptake of preventive healthcare.

More broadly, our findings show that migration-related inequalities intersect with gender-specific health determinants, increasing disparities in some areas of care (2, 41). Women typically use healthcare and preventive services more than men, which may make inequalities more apparent in this group (42) However, the proactive nature of preventive care makes it especially sensitive to barriers such as access, health literacy, and system navigation (35, 37).

In this context, immigrant women may face a double vulnerability. Migration-related barriers—such as language difficulties, limited system knowledge, and administrative obstacles—can limit access to preventive services (37). At the same time, gender-related factors, including caregiving roles, socioeconomic disadvantage, and cultural norms, may further reduce engagement, especially for non-urgent care (43). Evidence from Spain supports this view, showing that gender inequalities persist within immigrant groups, suggesting migration may reinforce existing social gradients (18, 19).

The role of language barriers as a possible explanation for the larger disparities observed among immigrant women is relevant (44). Unfortunately, the SNHS does not collect information on participants' Spanish-language proficiency, preventing us from directly examining this hypothesis. In some contexts, immigrant men may have greater exposure to Spanish through employment, whereas immigrant women may experience greater social isolation or be concentrated in domestic and caregiving roles that provide fewer opportunities for language practice.

From a policy perspective, our findings suggest that achieving equitable healthcare utilization among immigrant populations requires interventions that extend beyond universal free healthcare coverage (26). Common priorities include improving health literacy, multilingual communication, culturally responsive care, and patient navigation, while reducing organizational, administrative, and indirect socioeconomic barriers that may limit access to healthcare. Routine monitoring of healthcare utilization by migration status would also help identify persistent gaps and evaluate the effectiveness of equity-oriented interventions (26).

Our findings also highlight the need for service-specific strategies. For specialist care, strengthening coordination between primary and specialist care, improving referral completion, and reducing public-sector waiting times may help reduce inequalities (9). In mental healthcare, expanding publicly funded psychological services within primary care and community settings, together with culturally adapted care and professional interpretation, could improve access (10, 29–31). In contrast, the marked disparities observed in dental care reinforce the need to reduce financial barriers by expanding publicly funded dental services (15, 16, 34). Finally, the persistent inequalities in preventive screening among immigrant women support the implementation of proactive, culturally tailored programs incorporating multilingual invitations and reminders, community outreach, patient navigation, and flexible delivery models to improve participation (35–40).

A major strength of this study is its comprehensive and contemporary assessment of healthcare inequalities in Spain. Using the most recent nationally representative health survey, we examined a broad range of healthcare services across the continuum of care, including publicly funded services, predominantly privately delivered services such as dental care, physiotherapy, and psychological care, and multiple preventive activities. The matched, sex-stratified design and extensive adjustment for sociodemographic characteristics, comorbidities, lifestyle factors, and self-rated health further strengthened the analysis by incorporating information that is often unavailable in clinical or administrative databases (45, 46).

Several limitations should be noted. The cross-sectional design precludes causal inference, as exposure and outcome are measured simultaneously (47). All variables were self-reported and may be affected by recall bias or misclassification, potentially introducing measurement error (48–50). Key migration variables (e.g., length of residence, legal status, country of origin) were unavailable, limiting exploration of heterogeneity. This may affect employment status, as the survey does not capture informal or undeclared employment. If informal employment is more frequent among some immigrant groups, particularly in occupations such as domestic or caregiving work, some participants may have been misclassified according to their employment status. This potential misclassification could have influenced the estimated associations. Furthermore, as previously mentioned, the SNHS does not provide sufficiently detailed information on immigrants' language preference or proficiency to explore this important factor (44). Even if participants were exactly matched by autonomous community of residence to minimize regional variation in socioeconomic context and healthcare organization, residual heterogeneity within autonomous communities could not be accounted for. Large autonomous communities include populations with substantial socioeconomic diversity, and some unmeasured contextual factors may therefore have influenced healthcare utilization. Finally, despite adjustment and matching, residual confounding cannot be excluded.

In conclusion, immigrant populations in Spain continue to show lower utilization of healthcare services and lower participation in preventive activities compared with native born individuals, although many of these differences were partly attenuated after adjustment for sociodemographic characteristics, comorbidities, and lifestyle factors, several disparities remained statistically significant. Disparities are not uniform across the healthcare system, but are concentrated in specialist care and preventive services, and are particularly pronounced among women. These findings suggest that multiple factors contribute to healthcare inequalities among immigrant populations, although the relative contribution of these determinants cannot be established using the present cross-sectional design. From a public health perspective, ensuring equity therefore requires interventions that go beyond universal coverage, combining efforts to reduce structural and informational barriers with targeted, culturally sensitive strategies aimed at improving engagement with preventive services and continuity of care, particularly among immigrant women.

## Data Availability

Publicly available datasets were analyzed in this study. This data can be found here: https://www.sanidad.gob.es/estadisticas/microdatos.do.

## References

[1] Rechel B Mladovsky P Ingleby D Mackenbach JP McKee M. Migration and health in an increasingly diverse Europe. Lancet. (2013) 381:1235–45. doi: 10.1016/S0140-6736(12)62086-8 23541058

[2] Lebano A Hamed S Bradby H Gil-Salmerón A Durá-Ferrandis E Garcés-Ferrer J . Migrants‘ and refugees' health status and healthcare in Europe: a scoping literature review. BMC Public Health. (2020) 20:1039. doi: 10.1186/s12889-020-08749-8 32605605 PMC7329528

[3] Sarría-Santamera A Hijas-Gómez AI Carmona R Gimeno-Feliú LA. A systematic review of the use of health services by immigrants and native populations. Public Health Rev. (2016) 37:28. doi: 10.1186/s40985-016-0042-3 29450069 PMC5810113

[4] Neureiter M Waldmann G. Migration and Health Inequalities in Europe: Examining the Impact of Discriminatory Climates on Migrants' Unmet Healthcare Needs. Int Migration & Integration. (2026) 27:213–234. doi: 10.1007/s12134-025-01292-8

[5] Norredam M Nielsen SS Krasnik A. Migrants' utilization of somatic healthcare services in Europe–a systematic review. Eur J Public Health. (2010) 20:555–63. doi: 10.1093/eurpub/ckp195 20040522

[6] Carrasco-Garrido P De Miguel AG Barrera VH Jiménez-García R. Health profiles, lifestyles and use of health resources by the immigrant population resident in Spain. Eur J Public Health. (2007) 17:503–7. doi: 10.1093/eurpub/ckl279 17251304

[7] Bertakis KD Azari R Helms LJ Callahan EJ Robbins JA. Gender differences in the utilization of health care services. J Fam Pract. (2000) 49:147–52. 10718692

[8] Garcia-Subirats I Vargas I Sanz B Malmusi D Ronda E Ballesta M . Changes in access to health services of the immigrant and native-born population in Spain in the context of economic crisis. Int J Environ Res Public Health. (2014) 11:10182–201. doi: 10.3390/ijerph111010182 25272078 PMC4210974

[9] Villarroel N Artazcoz L. Different Patterns in Health Care Use Among Immigrants in Spain. J Immigr Minor Health. (2016) 18:318–29. doi: 10.1007/s10903-015-0202-4 25862208

[10] Gotsens M Malmusi D Villarroel N Vives-Cases C Garcia-Subirats I Hernando C . Health inequality between immigrants and natives in Spain: the loss of the healthy immigrant effect in times of economic crisis. Eur J Public Health. (2015) 25:923–9. doi: 10.1093/eurpub/ckv126 26136466

[11] Santamaría Rodríguez S Martínez Negro I Jiménez Viseu Pinheiro I Pulido Manzanero J Ares Blanco S. Acceso al sistema sanitario de la población inmigrante en España según posición socioeconómica. Revista Clí*nica de Medicina de Familia*. (2025) 18:21–8. doi: 10.55783/rcmf.180105

[12] Henares-Montiel J Ruiz-Perez I Mendoza-Garcia O. Health inequalities between male and female immigrants in Spain after the beginning of the economic crisis. Health Soc Care Community. (2018) 26:891–7. doi: 10.1111/hsc.12613 30014605

[13] Gimeno-Feliu LA Pastor-Sanz M Poblador-Plou B Calderón-Larrañaga A Díaz E Prados-Torres A. Overuse or underuse? Use of healthcare services among irregular migrants in a North-Eastern Spanish región. Int J Equity Health. (2021) 20:41. doi: 10.1186/s12939-020-01373-3 33472644 PMC7816492

[14] Dania Rocío DR Valentín HB Isabel JT Pilar CG. Factors Associated to Medication Consumption Among the Immigrant Population Residing in Spain. J Immigr Minor Health. (2018) 20:909–19. doi: 10.1007/s10903-017-0608-2 28597232

[15] Zuniga-Jara S Zuniga-Soria M Ruiz-Campo S. and Soria-Barreto K. Socioeconomic inequalities in unmet dental care needs in Spain. Front Oral Health. (2026) 7:1768458. doi: 10.3389/froh.2026.1768458 42078329 PMC13134389

[16] Agudelo-Suárez AA Muñoz-Pino N Vivares-Builes AM Ronda-Pérez E. Oral Health and Oral Health Service Utilization in Native and Immigrant Population: A Cross-Sectional Analysis from the PELFI Cohort in Spain. J Immigr Minor Health. (2020) 22:484–93. doi: 10.1007/s10903-020-00972-0 31919785

[17] Hidalgo IR Pisano-González MM García CF Bywall KS Andersson SW Avagnina B . IDEAHL consortium. Digital health literacy and immigrants' access to digitalized healthcare: a comparative study of Spain, and Sweden - implications for public health policy. BMC Public Health. (2026) 26:660. doi: 10.1186/s12889-026-26380-x 41593594 PMC12918215

[18] Pérez-Urdiales I. Undocumented immigrants' and immigrant women's access to healthcare services in the Basque Country (Spain). Glob Health Action. (2021) 14:1896659. doi: 10.1080/16549716.2021.1896659 33975531 PMC8118419

[19] Gea-Sánchez M Alconada-Romero Á Briones-Vozmediano E Pastells R Gastaldo D Molina F. Undocumented Immigrant Women in Spain: A Scoping Review on Access to and Utilization of Health and Social Services. J Immigr Minor Health. (2017) 19:194–204. doi: 10.1007/s10903-016-0356-8 26880030

[20] Fagundo-Rivera J García-Lozano MS Portero-Prados FJ Romero-Castillo R Badillo-Sánchez N Fernández-León P. Barriers to healthcare access for irregular immigrants after their arrival in Spain: a systematic review. Eur J Public Health. (2025) 35:407–22. doi: 10.1093/eurpub/ckaf042 40204625 PMC12192432

[21] Medina P Maia AC Costa A. Health Literacy and Migrant Communities in Primary Health Care. Front Public Health. (2022) 9:798222. doi: 10.3389/fpubh.2021.798222 35141189 PMC8818741

[22] Encuesta de Salud de España 2023. Metodologia. Available online at: https://www.sanidad.gob.es/en/estadEstudios/estadisticas/encuestaSaludEspana/ESdE2023/ESdE23_Metodologia.pdf (accessed 29 April 2026)

[23] Encuesta de Salud de España - ESdE 2023. Available online at: https://www.sanidad.gob.es/estadEstudios/estadisticas/encuestaSaludEspana/home.html (accessed 29 April 2026)

[24] Macias-Konstantopoulos WL Collins KA Diaz R Duber HC Edwards CD Hsu AP . Race, Healthcare, and Health Disparities: A Critical Review and Recommendations for Advancing Health Equity. West J Emerg Med. (2023) 24:906–18. doi: 10.5811/WESTJEM.58408 37788031 PMC10527840

[25] Thorpe RJ Jr Bruce MA Wilder T Jones HP Thomas Tobin C Norris KC. Health Disparities at the Intersection of Racism, Social Determinants of Health, and Downstream Biological Pathways. Int J Environ Res Public Health. (2025) 22:703. doi: 10.3390/ijerph22050703 40427819 PMC12111065

[26] Ministerio de Sanidad Servicios Sociales e Igualdad. Comisión para reducir las Desigualdades sociales en salud en España. Avanzando hacia la equidad. Madrid: Ministerio de Sanidad, Servicios sociales e Igualdad (2015). 95p. Disponible en: Available online at: https://www.sanidad.gob.es/areas/promocionPrevencion/promoSaludEquidad/equidadYDesigualdad/estrategia/docs/Propuesta_Politicas_Reducir_Desigualdades.pdf

[27] Graetz V Rechel B Groot W Norredam M Pavlova M. Utilization of health care services by migrants in Europe-a systematic literature review. Br Med Bull. (2017) 121:5–18. doi: 10.1093/bmb/ldw057 28108435

[28] Morris S Sutton M Gravelle H. Inequity and inequality in the use of health care in England: an empirical investigation. Soc Sci Med. (2005) 60:1251–66. doi: 10.1016/j.socscimed.2004.07.016 15626522

[29] Mason J Laporte A McDonald JT Kurdyak P Fosse E de Oliveira C. Assessing the “healthy immigrant effect” in mental health: Intra- and inter-cohort trends in mood and/or anxiety disorders. Soc Sci Med. (2024) 340:116367. doi: 10.1016/j.socscimed.2023.116367 38039769

[30] Bianucci R Charlier P Perciaccante A Lippi D Appenzeller O. The “Ulysses syndrome”: An eponym identifies a psychosomatic disorder in modern migrants. Eur J Intern Med. (2017) 41:30–2. doi: 10.1016/j.ejim.2017.03.020 28377064

[31] Bauldry S Szaflarski M. Immigrant-based Disparities in Mental Health Care Utilization. Socius. (2017) 3:10.1177/2378023116685718. doi: 10.1177/2378023116685718 PMC5568671 28845455

[32] Mohammadifirouzeh M Oh KM Basnyat I Gimm G. Factors Associated with Professional Mental Help-Seeking Among US Immigrants: A Systematic Review. J Immigr Minor Health. (2023) 25:1118–36. doi: 10.1007/s10903-023-01475-4 37000385 PMC10063938

[33] Bilican S Irfan M Cox A Salaets H Sabbe M Schoenmakers B. Access to mental healthcare for refugees, asylum seekers and migrants: an umbrella review of barriers. BMJ Open. (2025) 15:e096267. doi: 10.1136/bmjopen-2024-096267 PMC12161349 40484433

[34] López-López S Del Pozo-Rubio R Ortega-Ortega M Escribano-Sotos F. Catastrophic out-of-pocket payments for dental treatment: regional evidence from Spain. BMC Health Serv Res. (2023) 23:784. doi: 10.1186/s12913-023-09761-5 37480038 PMC10362549

[35] Dumky H Fridell K Leifland K Metsälä E. Breast cancer screening and immigrant women-A scoping review of attendance, knowledge, barriers and facilitators. Nurs Open. (2023) 10:5843–56. doi: 10.1002/nop2.1865 37259178 PMC10416041

[36] Restivo V Graci D Immordino A Mancuso DG Morales F Pace C . Cervical Cancer Screening in Refugee and Migrant Populations: Results of Systematic Review and Meta-Analysis in Cross-Sectional and Cohort Studies. Cancers. (2025) 17:2966. doi: 10.3390/cancers17182966 41008810 PMC12468638

[37] Khatri RB Endalamaw A Darssan D Assefa Y A. scoping review of the levels, implementation strategies, enablers, and barriers to cervical, breast, and colorectal cancer screening among migrant populations in selected English-speaking high-income countries. PLoS ONE. (2025) 20:e0329854. doi: 10.1371/journal.pone.0329854 40811730 PMC12352849

[38] Ola I Cardoso R Hoffmeister M Brenner H. Utilization of colorectal cancer screening tests across European countries: a cross-sectional analysis of the European health interview survey 2018-2020. Lancet Reg Health Eur. (2024) 41:100920. doi: 10.1016/j.lanepe.2024.100920 38707865 PMC11067466

[39] Fabiani M Riccardo F Di Napoli A Gargiulo L Declich S Petrelli A. Differences in Influenza Vaccination Coverage between Adult Immigrants and Italian Citizens at Risk for Influenza-Related Complications: A Cross-Sectional Study. PLoS ONE. (2016) 11:e0166517. doi: 10.1371/journal.pone.0166517 27832186 PMC5104396

[40] Allegri C Belgiojoso EBD Rimoldi SML. Immigrants' self-perceived barriers to healthcare: A systematic review of quantitative evidence in European countries. Health Policy. (2025) 154:105268. doi: 10.1016/j.healthpol.2025.105268 39983630

[41] Vidal EM Wickramage KP. Tracking migration and health inequities. Bull World Health Organ. (2024) 102:143–5. doi: 10.2471/BLT.23.290776 38313145 PMC10835634

[42] Golinelli D Sanmarchi F Guarducci G Palombarini J Benetti P Rosa S . Gender differences in healthcare utilization across Europe: Evidence from the European Health Interview Survey. Health Policy. (2025) 162:105448. doi: 10.1016/j.healthpol.2025.105448 41022014

[43] Ouanhnon L Astruc P Freyens A Mesthé P Pariente K Rougé D . Women's health in migrant populations: a qualitative study in France. Eur J Public Health. (2023) 33:99–105. doi: 10.1093/eurpub/ckac133 36130410 PMC9897994

[44] Steinberg EM Valenzuela-Araujo D Zickafoose JS Kieffer E DeCamp LR. The “Battle” of Managing Language Barriers in Health Care. Clin Pediatr (Phila). (2016) 55:1318–27. doi: 10.1177/0009922816629760 26896341 PMC4990509

[45] Wan F Colditz GA Sutcliffe S. Matched Versus Unmatched Analysis of Matched Case-Control Studies. Am J Epidemiol. (2021) 190:1859–66. doi: 10.1093/aje/kwab056 33693492 PMC8681061

[46] Kuo CL Duan Y Grady J. Unconditional or Conditional Logistic Regression Model for Age-Matched Case-Control Data? Front Public Health. (2018) 6:57. doi: 10.3389/fpubh.2018.00057 29552553 PMC5840200

[47] Savitz DA Wellenius GA. Can Cross-Sectional Studies Contribute to Causal Inference? It Depends Am J Epidemiol. (2023) 192:514–6. doi: 10.1093/aje/kwac037 35231933

[48] Baumeister SE Kocher T Papapanou PN Holtfreter B Demmer RT. Cross-Sectional Studies: Strengths, Limitations, and Methodological Considerations. J Periodontal Res. (2026). doi: 10.1111/jre.70063 41487096 PMC13665586

[49] Brusco NK Watts JJ. Empirical evidence of recall bias for primary health care visits. BMC Health Serv Res. (2015) 15:381. doi: 10.1186/s12913-015-1039-1 26373712 PMC4572632

[50] Althubaiti A. Information bias in health research: definition, pitfalls, and adjustment methods. J Multidiscip Healthc. (2016) 9:211–7. doi: 10.2147/JMDH.S104807 27217764 PMC4862344

