# Supplementary material for: Sex-specific differences in healthcare utilization and preventive service uptake between immigrant and native-born populations in Spain: a matched analysis of the 2023 national health survey
Source: Front Public Health. 2026 Aug 5;14:1894084. doi: 10.3389/fpubh.2026.1894084 (PMC13486252; doi:10.3389/fpubh.2026.1894084)
Supplement: Supplementary file 1 [file Table_1.docx]

**Supplementary Table 1**. Definition of variables according to the questions included in the Spanish National Health Interview Survey 2023.

| **Questions** | | **Description and answer** | | **Variables name** | | **Categories** | |
| --- | --- | --- | --- | --- | --- | --- | --- |
| Which is your gender? | 1. Male  2. Female | | Gender | | 1. Male  2. Female | |  |
| Age in years | Age groups | |  | |  | |  |
| What is your country of birth? | 1. Spain  2. Other | | Country of birth | | 1. Spain  2. Abroad | |  |
| What level of education have you completed? | 1. Does not know how to read or write  2. Incomplete primary education  3. Complete primary education  4. First stage of Secondary Education, with or without a qualification  5. Elementary Spanish Upper Secondary Education  6. Upper secondary education  7. Intermediate vocational training or equivalent  8. Advanced vocational training or equivalent  9. University studies or equivalent  10. University (master, PhD….) | | Educational level | | 1. Ended before 13 years of age: Options 1 to 3  2. Ended from 13 to 16 years: Options 4 and 5  3. Ended from 16 to 18 or vocational training. Options 6 to 8.  4 Completed university studies: Options 9 and 10 | |  |
| Which is your employment status? | 1. Working 2. Unemployed 3. Retired 4. Studying 5. Unable to work 6. Housework 7. Other | | Employment status | | 1. Working. Option 1  2. Unemployed Option 2  3. Retired, or disable Options 3 and 5  4. Housework or studying. Options 4 and 6 | |  |
| Of the following options, which describes your current situation? | 1. Living with your spouse  2. Living with a common-law partner  3. Not living together as a couple | | Living with a partner | | 1. Yes: Option 1 and 2  2. No: option 3 | |  |
| What type of health insurance coverage do you have? | Respondents were classified according to their type of health insurance coverage | | Private health insurance coverage | | 1. Yes  2. No | |  |
| In the past twelve months, how is your perception of your general health status? | 1. Very good  2. Good  3. Fair  4. Bad  5. Very bad | | Self-rated health | | 1. Very good/good: Options 1 and 2  2. Fair/poor/very poor: Options 3 to 5 | |  |
| Do you have any chronic or long-term health problems or diseases? We understand for a long duration if the health problem or disease has lasted or is expected to last 6 months or longer. | 1. Yes  2. No | | Chronic conditions | | 1. Yes  2. No | |  |
| Has your doctor told you that you are suffering from high blood pressure? | 1. Yes  2. No | | High blood pressure | | 1. Yes 2. No | |  |
| “Has your doctor told you that you are suffering from…?”   - Myocardial infarction - Angina/coronary. - Heart failure - Other heart diseases - Stroke | Respondents reporting any of the listed cardiovascular conditions were classified as having heart diseases and stroke | | Heart diseases and stroke | | 1. Yes (any item = Yes)  2. No (all items = No) | |  |
| “Has your doctor told you that you are suffering from…?”   - Osteoarthritis (excluding arthritis); - Chronic cervical back pain - Chronic lumbar back pain - Osteoporosis | Respondents reporting any of the listed musculoskeletal conditions were classified as having musculoskeletal disorders | | Musculoskeletal disease | | 1. Yes (any item = Yes)  2. No (all items = No) | |  |
| “Has your doctor told you that you are suffering from…?”   - Asthma - Chronic bronchitis/emphysema/COPD | Respondents reporting any of the listed respiratory conditions were classified as having respiratory disease | | Respiratory diseases | | 1. Yes (any item = Yes)  2. No (all items = No) | |  |
| “Has your doctor told you that you are suffering from…?”   1. Gastric or duodenal ulcer 2. Chronic constipation  - Cirrhosis/liver dysfunction | Respondents reporting any of the listed gastrointestinal conditions were classified as having chronic gastrointestinal disorder | | Chronic gastrointestinal disorder | | 1. Yes (any item = Yes)  2. No (all items = No) | |  |
| “Has your doctor told you that you are suffering from…?”   - Alzheimer’s disease or other dementias. - Migraine or frequent headache | Respondents reporting any of the listed neurological conditions were classified as having neurological disorder | | Neurological disorder | | 1. Yes (any item = Yes)  2. No (all items = No) | |  |
| “Has your doctor told you that you are suffering from…?”   - Depression - Anxiety - Other mental health problems | Respondents reporting any of the listed mental health conditions were classified as having mental health disorder | | Mental health disorders | | 1. Yes (any item = Yes)  2. No (all items = No) | |  |
| Has your doctor told you that you are suffering from malignant tumors? | Respondents reporting a history of malignant tumors | | Cancer | | 1. Yes  2. No | |  |
| Has your doctor told you that you are suffering from diabetes? | Respondents reporting a history of diabetes | | Diabetes | | 1. Yes  2. No | |  |
| During the past 12 months, how often have you had alcoholic beverages of any kind (i.e. beer, wine, spirits, distilled and mixed drinks, or other alcoholic beverages)? | 1. Daily or almost daily  2. 5-6 days per week  3. 3-4 days per week  4. 1-2 days per week  5. 2-3 days in a month  6. Once a month  7. Less than once a month  8. Not in the last 12 months have I stopped drinking  9. Never or just a few sips to taste it throughout life | | Alcohol use | | 1. Daily or weekly Options 1 to 4  2. Few times a year Options 5 to 7  3 Not last year or eve. Options 8 and 9 | |  |
| Could you tell me if you smoke? | 1. Yes, I smoke daily  2. Yes, I smoke, but not daily  3. I don't currently smoke but have smoked before  4. I neither smoke nor have I ever smoked regularly | | Tobacco use | | 1. Currently Options 1 and 2  2. Ex-smoker: Options 3  3. Never smoked. Option 4 | |  |
| Which of these possibilities best describes how often you do some physical activity in your free time? | 1. I don't exercise. I occupy my free time almost completely sedentary.  2. I do some occasional physical or sports activities  3. I do physical activity several times a month  4. I do sports or physical training several times a week | | Physically active | | 1. No: Option 1  2. Yes: Option 2 to 4 | |  |
| 1. Could you tell me how tall you are, approximately, without shoes?  2. Could you tell me your weight, approximately, without shoes and clothes? | Body mass index is calculated with the formulae:  Weight in kg/ (Height in meters) | | Body mass index | | 1. Normal (<25)  2. Overweight (25-29.9)  3.Obesity ( ≥30) | |  |
| When was the last time you consulted your primary care doctor? | 1. In the last 4 weeks  2. Between 4 weeks and 12 months .  3. 12 months or more ago  4. Never | | Primary care doctor visit in last 12 months | | 1. Yes. Options 1 and 2.  2. No. Options 3 and 4 | |  |
| When was the last time you consulted a specialist doctor? | 1. In the last 4 weeks  2. Between 4 weeks and 12 months.  3. 12 months or more ago  4. Never | | Specialist doctor visit in last 12 months | | 1. Yes. Options 1 and 2.  2. No. Options 3 and 4 | |  |
| In the last 12 months, have you visited a psychologist? | 1.Yes  2.No | | Visit to psychologist | | 1. Yes  2. No | |  |
| In the last 12 months, have you visited a physiotherapist? | 1.Yes  2.No | | Visit to physiotherapist | | 1. Yes  2. No | |  |
| When was the last time you visited the dentist or dental hygienist? | 1. In the last 3 months  2. Between 3 months and 6 months.  3. Between 6 months and 12 months.  4. 12 months or more ago  5. Never | | Dentist last 12 months | | 1. Yes. Options 1 to 3.  2. No. Options 4 and 5 | |  |
| In the last 12 months, have you visited the emergency room? | 1. Yes  2. No | | Emergency room visit in last 12 months | | 1. Yes  2. No | |  |
| In the last 12 months, have you had to be hospitalized for at least one night? | 1.Yes  2.No | | Hospital admission | | 1. Yes  2. No | |  |
| Uptake of colorectal cancer screening. It was determined by asking:  ‘Have you ever undergone a fecal occult blood test?’. Those who answered affirmatively were asked a second question, ‘When was the last time you had a fecal occult blood test? | 1. In the past 12 months  2. Over 1 year but less than 2 years ago  3. Over 2 years but less than 3 years ago  4. Over 3 years but less than 5 years ago  5. Over 5 years ago | | FOBT | | 1. Yes: Options 1 and 2  2. No: Options 3 to 5 | |  |
| Uptake of breast cancer screening. It was determined by asking:  ‘Have you ever undergone a mammogram?’. Those who answered affirmatively were asked a second question, "When was the last time you had a mammogram?” | 1. In the last 12 months  2. Over 1 year but less than 2 years ago  3. Over 2 years but less than 3 years ago  4. Over 3 years ago | | Mammogram | | 1. Yes: Options 1 to 3  2. No: Option 4 | |  |
| Uptake of cervical cancer screening. It was determined by asking:  ‘Have you ever undergone cervical cytology?’.  Women who answered affirmatively were asked,  ‘When was the last time you underwent cervical cytology? | 1. In the last 12 months  2. Over 1 year but less than 2 years ago  3. Over 2 years but less than 3 years ago  4. Over 3 years ago | | Pap Smear | | 1. Yes: Options 1 to 3  2. No: Option 4 | |  |
| Were you vaccinated against influenza during the last vaccination campaign? | 1.Yes  2.No | | Influenza vaccination | | 1.Yes  2. No | |  |
| Have you ever had your blood cholesterol measured in last 12 months? | 1.Yes  2.No | | Blood Cholesterol | | 1.Yes  2.No | |  |
| Have your blood sugar ever been checked by a healthcare professional? | 1.Yes  2.No | | Blood Sugar | | 1.Yes  2.No | |  |
